# Do Overcrowding and Turnover Cause Violence in Prison?

**DOI:** 10.3389/fpsyt.2019.01015

**Published:** 2020-01-24

**Authors:** Stéphanie Baggio, Nicolas Peigné, Patrick Heller, Laurent Gétaz, Michael Liebrenz, Hans Wolff

**Affiliations:** ^1^Division of Prison Health, Geneva University Hospitals and University of Geneva, Geneva, Switzerland; ^2^Department of Forensic Psychiatry, Institute of Forensic Medicine, University of Bern, Bern, Switzerland; ^3^Adult Psychiatry Division, Department of Mental Health and Psychiatry, Geneva University Hospitals, Geneva, Switzerland; ^4^Division of Tropical and Humanitarian Medicine, Geneva University Hospitals and University of Geneva, Geneva, Switzerland

**Keywords:** health policy, forensic, institutional factor, misconduct, public health, prison

## Abstract

Violence is common in prison and its individual risk factors are well documented. However, there is a mixed evidence on the relationship between prison violence and institutional factors, such as overcrowding and turnover, and recent research suggested that these factors may not be important or relevant. This study investigated the association between prison violence and institutional factors in a Swiss pre-trial prison between 2013 and 2018. Measures included violence (assaults requiring immediate medical attention) as well as the annual overcrowding and turnover rates. Using a meta-regression, the results showed that prison violence was higher when overcrowding and turnover increased. Overall, our study highlighted that institutional prison factors might have notable detrimental effects on prison life. Reduction of prison overcrowding and turnover appear critical to reduce prisoners’ vulnerability. Turning prison into safe places designed to promote desistance would probably not be achievable without considering these crucial factors.

## Introduction

Prison overcrowding, when the number of prisoners exceeds the prison capacity, is an important concern worldwide. In 2018, overcrowding remained one of the most important issues in prison (1), with 27 countries operating at 150% to 200% (2). Turnover, the rate at which the prison population is renewed, has been less extensively studied (3, 4), but may also have detrimental consequences for prisoners (4). Both can undermine the ability of prison systems to meet human needs, including access to appropriate accommodation, timely health care, and access to rehabilitation programs and educational or vocational activities (5).

However, in a recent empirical study, Fazel, Ramesh & Hawton (3) underscored the importance of individual over institutional factors. In their multicentric study conducted in 24 high-income countries, there was no significant association between prison suicide and two major institutional factors, namely overcrowding and turnover. These findings resulted in a call to focus on individual and relevant ecological factors (3). This mixed evidence also applies to prison violence: A meta-analysis to conclude that future policies should focus on “more important predictors” than overcrowding to predict (violent) misconduct (6, p. 409), even if overcrowding has long been described as a potential risk for prison violence (7).

These conclusions have risen legitimate fears of misinterpretation and neglect of critical institutional factors (8). Besides, very recent prison studies highlighted significant associations between overcrowding, turnover, and self-harm (4); and between overcrowding and violent misconduct (9, 10). Another recent study also reported that institutional infractions were more likely to happen a few months after entry (11). As turnover is associated with an increased number of prison entries, it may lead to increased levels of misconduct, infractions, and violence.

This study focused on violence against others, as there is a paucity of empirical studies investigating the association between institutional factors and this kind of violence. Prison violence has been most often investigated using assaults registered in official prison records (i.e., “violent misconduct”) (6, 7). In addition, to our knowledge, previous studies on prison violence focused on overcrowding and turnover has been neglected. We hypothesized that institutional factors would lead to increased levels of violence, and thus, that these factors should not be neglected in empirical prison studies and health policy.

## Materials and Methods

### Setting

Prison-level data were collected between 2013 and 2018 in a Swiss pre-trial prison located in Geneva (Champ-Dollon). This prison is mainly a pre-trial prison, but there are also sentenced detainees. In this prison, prisoners spend 23 h a day in their cell. The prison capacity was 376 (with 22 additional places in 2017 and 2018). Nurses are present in the prison 24/7 in a prison medical unit. This prison has been repeatedly criticized by the European Committee for the Prevention of Torture and Inhuman or Degrading Treatment or Punishment (CPT) for chronic overcrowding and detention setting, including lack of activities (12). Data were collected using prison-level statistics and prison nurses’ records. Since we used anonymous quality control data, ethical approval was not required.

### Measures

#### Prison Overcrowding

The annual overcrowding rate was computed by dividing the annual mean daily population by the prison capacity. It was extracted from the statistics available each year for the whole prison, upon request to the direction of the prison.

#### Turnover Ratio

The turnover rate was computed using the number of releases divided by the number of entries plus the average prison population of the previous year (3). It was also extracted from the statistics available each year for the whole prison, upon request to the direction of the prison.

#### Violence

Nurses recorded systematically and anonymously each assault requiring medical attention immediately after its occurrence, in accordance with the guidelines of a previous study on prison violence, recommending a systematic statistical recording of routine data on prison violence, to standardize injury surveillance (13).

### Statistical Analyses

We tested the association between violence, overcrowding, and turnover using a fixed-effect multivariate meta-regression. Each year was considered as a separate sample (too few events to consider months as separate samples). Analyses were performed with R 3.5.1 (package metaphor 2.0.0).

## Results

Over the study period, the average rate of overcrowding was 175.4% and the turnover rate 73.2%. This meant that the prison was overcrowded, as the number of prisoners exceeded its official capacity (100%). However, there is no official definition of what constitutes overcrowding (5). The turnover rate was also high, with on average 73.2% of the prison population entirely reviewed each year. On average, there was 9.1% of cases of violence/population of inmates over the study period. The meta-analytic prevalence estimate for prison violence over the study period was 8.5% (95% confidence interval: 7.6%–9.3%).

**Figure 1:**
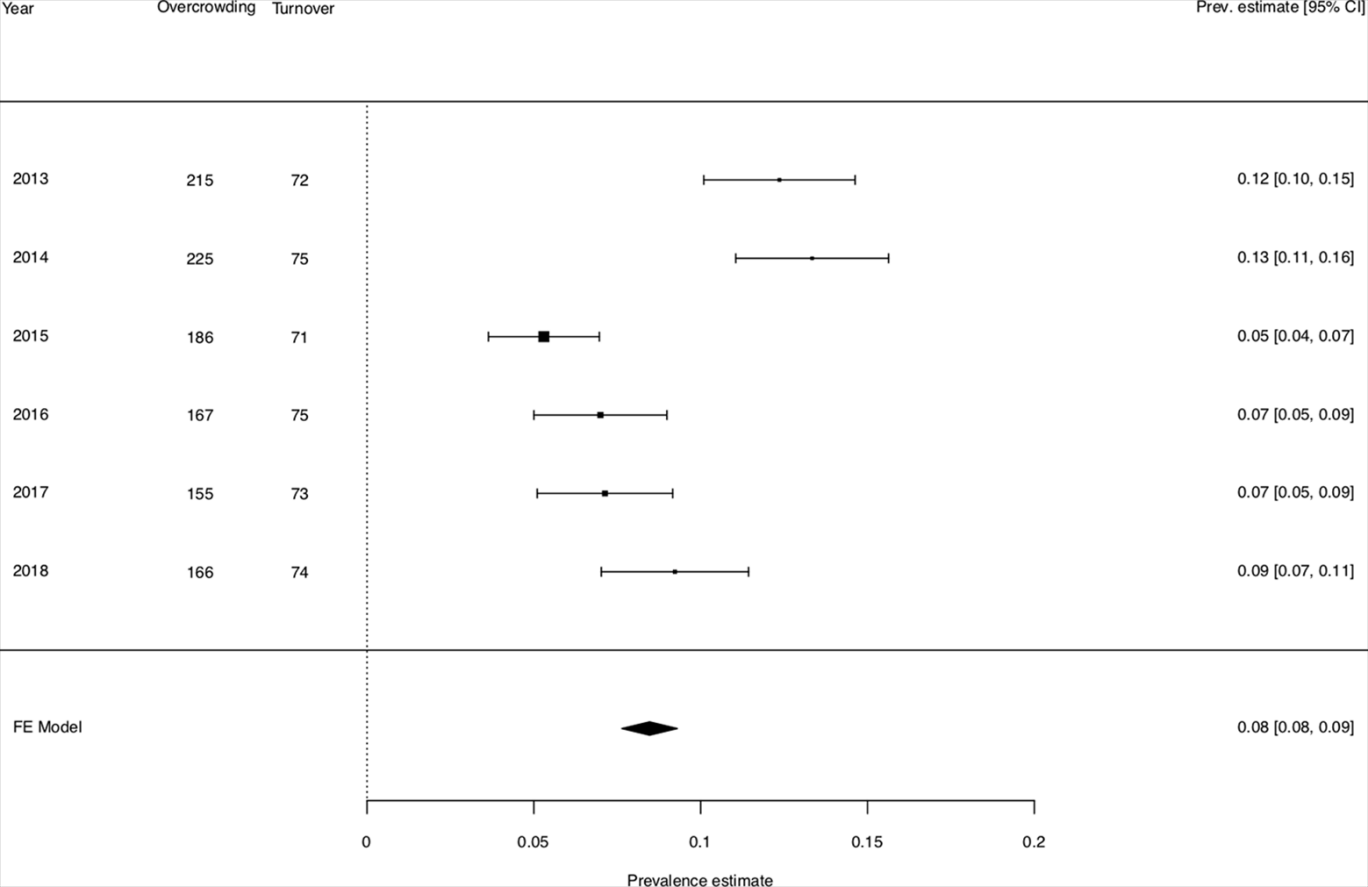
Forest plot of the effect of overcrowding and turnover on prison violence, sorted by overcrowding rate. 95% CI: 95% confidence intervals. Whiskers represent 95% CI for the prevalence estimate of each year. Prevalence estimates are reported for assaults requiring immediate medical attention. Overcrowding and turnover are reported as percentages.

There were significant effects of both overcrowding (b = 0.001, p < .001) and turnover (b = 0.009, p < .001) on prison violence. Increased overcrowding and turnover were associated with increased prevalence estimates of violence. When overcrowding increased of one point (on a one hundred percent scale), prison violence increased of 0.1 point of percentage. **Figure 1** shows that increased levels of overcrowding were associated with higher prevalence estimates of prison violence. When turnover increased of one point (on a one hundred percent scale), prison violence increased of 0.9 point of percentage. The pattern was less clear in the forest plot depicted in **Figure 1**, but the effect was nonetheless significant.

## Discussion

In our study, there was a meta-analytic percentage of 8.5% of assaults requiring immediate medical attention. This percentage ranged between previous estimates, from 0.8% for assaults classified as violent misconduct in official prison reports (9) to 23.5% of assaults (including assaults against staff) classified as disciplinary offences in official prison reports (10). As these studies used very different measures to assess prison violence and were conducted in different settings, comparisons are not possible.

Our study showed that institutional prison factors were significantly associated with prison violence (i.e., assaults requiring immediate medical attention). This result replicated recent empirical findings focusing on overcrowding in the US and using official misconduct reports (9, 10). Our study extended these results in a European country and with data not necessarily recorded in the official prison reports. It followed recent guidelines for systematic statistical recording of violence (13). In addition, to our knowledge, this study was the first to examine the association between turnover and prison violence, highlighting that this institutional factor also led to increased levels of prison violence.

Overall, our study highlighted that institutional prison factors might have notable detrimental effects on prison life and adjustment to prison life. Reduction of prison overcrowding and turnover appear critical to reduce prisoners’ vulnerability and they should not be neglected. These detrimental effects may be even worse for especially vulnerable people living in detention (e.g., those in bad health or having severe psychiatric disorders, older people). Even if these factors are not easily modifiable, future prison policies should be developed to promote prisoners’ health and rehabilitation. Indeed, (violent) misconduct is associated with increased recidivism (14).

Meanwhile, adequate prevention measures to reduce violence in overcrowded prisons are needed. It should include adequate occupational activities as well as screening and treatment for psychiatric disorders targeting specific needs; as well as enhancement of social skills, social relationships, and social support using relevant psychosocial programs (13, 15). Such need for adjustments in prison policy is regularly emphasized in the legal literature as well (16, 17).

This study has some limitations. A first limitation was the lack on individual data, such as personal risk factors for prison violence. However, the prison population of Champ-Dollon was stable over time [e.g., rates of psychiatric treatments and socio-demographic profiles, (18)] so we could be confident that the changes in prison violence was mostly related to the institutional factors. Second, the results were probably related to the specific characteristics of the prison, namely the lack of freedom of movement and activities. However, the 23-h confinement period per day and the lack of access to a workplace are comparable in most pre-trial prisons in Switzerland (17). Furthermore, Champ-Dollon is especially overcrowded (12). Another shortcoming was that we used an operationalization of prison violence (i.e., assaults requiring immediate medical attention) which did not allow comparisons with other studies. Our study missed less severe cases of violence (not requiring immediate medical care), but it used a less restrictive operationalization of prison violence in comparison with some previous studies relying exclusively on official prison reports. In addition, given its retrospective design, we were unable to collect information on violence against staff members. Future multicentric studies should include prisons’ characteristics, and especially time spent locked up in cells and available pro-health, pro-social, and occupational activities (4), as well as individual-level factors and all kinds of violence, including those against staff members. Further studies should also develop assessments of prison violence that allow comparisons between prisons and include less severe forms of violence. Finally, prison violence can also mean psychological violence, such as harassment, bullying, or sexual violence (19). Future studies should also investigate this kind of violence.

To conclude, we believe that institutional factors should not be neglected in prison research and future prison policies. Overcrowding and turnover have an important impact on prisoners’ health, prison life, and adjustment to prison life; even if these effects depend on the specific characteristics of the prison under study. Distress and misconduct in prison should be considered as the interplay between individual and institutional factor, and not only as something prisoners import in prison (8). Turning prison into safe places designed to promote desistance would probably not be achievable without considering these crucial factors.

## Data Availability Statement

The raw data supporting the conclusions of this article will be made available by the authors, without undue reservation, to any qualified researcher.

## Ethics Statement

Ethical review and approval was not required for the study on human participants in accordance with the local legislation and institutional requirements. Written informed consent for participation was not required for this study in accordance with the national legislation and the institutional requirements. Since we used anonymous quality control data, ethical approval was not required.

## Author Contributions

SB conceived the study’s objective, drafted the manuscript, and performed the statistical analyses. NP participated in data collection. PH, LG, ML, and HW made substantial contributions in the interpretation of the data. NP, PH, LG, ML, and HW revised the manuscript critically for important intellectual content. All authors approved the final version to be published and agreed to be accountable for all aspects of the work related to its accuracy and integrity.

## Conflict of Interest

The authors declare that the research was conducted in the absence of any commercial or financial relationships that could be construed as a potential conflict of interest.
